# Fc-Gamma Receptor 3B Copy Number Variation Is Not a Risk Factor for Behçet's Disease

**DOI:** 10.1155/2012/167096

**Published:** 2012-05-30

**Authors:** Rachel Black, Sue Lester, Emma Dunstan, Farhad Shahram, Abdolhadi Nadji, Noushin Bayat, Kayvan Saeedfar, Naghmeh Ziaei, Catherine Hill, Maureen Rischmueller, Fereydoun Davatchi

**Affiliations:** ^1^Department of Rheumatology, The Queen Elizabeth Hospital, 28 Woodville Road, Woodville South, SA, 5011, Australia; ^2^Rheumatology Research Centre, Tehran University of Medical Sciences, Shariati Hospital, Kargar Avenue, Tehran 14114, Iran; ^3^Rheumatology Department, Baqyiatallah University of Medical Sciences, Baghiatallah hospital, Molla Sadra Street, Tehran 14359, Iran; ^4^Chronic Respiratory Diseases Research Centre, Shahid Beheshti University of Medical Sciences, Massih Daneshvari Hospital, Shaid Bahonar Street, Tehran 19556, Iran; ^5^Discipline of Medicine, The University of Adelaide, Adelaide, SA 5005, Australia

## Abstract

Behçet's disease (BD) is an immune-mediated systemic vasculitis associated with HLAB51. Other gene associations are likely and may provide further insight into the pathogenesis of this disease. Fc-gamma receptors play an important role in regulating immune function. Copy number variation (CNV) of the Fc-gamma receptor 3B (FCGR3B) gene is associated with other inflammatory conditions and may also play a role in BD. The aim of this study was to determine whether CNV of the FCGR3B gene is associated with BD or its clinical features. FCGR3B copy number was determined for 187 Iranian patients and 178 ethnicity-matched controls using quantitative real-time PCR. The genotype frequencies were comparable in both BD patients and controls. The odds ratio for low copy number (<2CN) was 0.6 (*P* = 0.16) and the odds ratio for high copy number (>2CN) was 0.75 (*P* = 0.50). There was no association found between high or low CN of the FCGR3B gene and BD or its clinical features in this Iranian population. We are the first to report this finding which, when looked at in the context of other genetic studies, gives us further insight into the complex pathogenesis of BD.

## 1. Background

Behçet's disease (BD) is an immune-mediated, systemic vasculitis, in which blood vessels of all sizes (small, medium, and large) in both the venous and arterial circulation can be affected. Clinically it is characterised by recurrent aphthous ulcers of the mouth and/or genitals in combination with other systemic manifestations involving the skin, eyes, joints, vessels, gastrointestinal tract, or central nervous system [1].

The pathogenesis of BD is not understood. It lies at the crossroad between autoimmune and autoinflammatory disorders [2], may be triggered by infectious agents, and is characterised by a number of immunological aberrations, such as neutrophil hyperactivity (reviewed in [3, 4]). There is also clearly a genetic component. BD is most frequently observed in populations around the Mediterranean basin, the Middle East, and the Far East, and the clustering of BD in populations along the ancient Silk Route suggests that an inherited tendency to BD was spread by travellers along these trading routes. Multiple studies, in multiple populations, have confirmed a strong association between HLA-B51 and BD [4], but other genes are also likely to be involved. Copy number variation in the *FCGR3B *gene is one candidate gene of interest.

Copy number variation (CNV) is departure from the normal diploid number of genes (*n* = 2) which may arise from gene duplication and deletion events and which may contribute substantially to quantitative variation in expression. An increasing number of CNVs have been characterised in the human genome with implications for both evolution and disease susceptibility [5]. CNV has been well characterised in the FCGR gene cluster on chromosome 1q23. This cluster carries 5 highly homologous genes that encode for low affinity receptors for IgG-complexed antigens, which are expressed widely throughout the haematopoietic system. These low affinity Fc-gamma receptors are involved in the regulation of a multitude of innate and adaptive immune responses, with implications for both response to infection and susceptibility to autoimmunity [6].

CNV in the *FCGR3B* gene is of particular interest. FCGR3B is expressed almost exclusively on neutrophils [6], and there is a clear correlation between gene copy number and FCGR3B cell surface expression, neutrophil adherence to IgG-coated surfaces, and immune complex uptake [7]. Further, multiple studies have identified low FCGR3B CN (i.e., <2 copies) as a risk factor for systemic autoimmune diseases, such as systemic lupus erythematosus [8–10], rheumatoid arthritis [11, 12], and primary Sjögren's syndrome [9, 10]. We therefore examined the association between *FCGR3B* CNV and BD, in a cohort of Iranian patients.

## 2. Methods

### 2.1. Subjects

Ethics approval was obtained from the Rheumatology Research Centre's ethics committee of Tehran University of Medical sciences. A written informed consent was obtained from all participants. 187 Iranian BD patients were recruited from the Behçet's clinic at Shariati Hospital, Tehran, from early 2005 until late 2006. 178 ethnically-matched, unrelated healthy volunteers were also recruited as normal controls between 2005 and 2007. All the BD patients recruited for this study met the International Study Group (ISG) criteria for BD [13]. Baseline data was collected using a standardised questionnaire at the time of blood sampling. Information was obtained regarding their age, gender, ethnicity, clinical features, and family history. Serotyping for HLA-B51 status was carried out in selected general immunologic laboratories within Iran using routine commercial kits. Samples were then transported to Australia for further analysis.

### 2.2. FCGR3B Copy Number (CN) Genotyping

Genomic FCGR3B CN was determined using a quantitative real-time PCR method, as previously described [11]. Briefly, a duplex Taqman copy number assay was performed, using FCGR3B specific primers (Applied Biosystems, Hs04211858, FAM-MGB dual labeled probe) and RNase P (Applied Biosystems, product 4403326, VIC-TAMRA dual-labeled probe) as the reference assay. The assay was performed according to the manufacturer's instructions and PCR reactions were run on an Applied Biosystems 7300 Real-Time PCR machine. All samples were tested in triplicate, and fluorescence signals were normalised to ROX. Copy number was determined using Copy Caller software (v.1.0, Applied Biosystems, USA), and results were accepted only when calling confidence was >80%, and ΔCq standard deviation between replicates was <0.20.

### 2.3. Statistical Analysis

Analysis of *FCGR3B* CN in BD patients compared to controls, and with clinical manifestations within BD patients, was performed using logistic regression. Analysis was performed using Statistica v6 (Statsoft, Tulsa OK, USA).

## 3. Results

187 BD patients were included in the study (mean age 32.7 ± 8.2, 48% female). Baseline characteristics are summarised in Table 1. Of those who were tested for HLA status, 51% (89 of 175) were HLA B5 positive and 51% (41 of 80) were HLA B51 positive.

The frequency of *FCGR3B* CN variants in both BD patients and controls is presented in Table 2. Copy numbers ranged from 1 to 4, and no null genotypes were observed in this cohort. The genotype frequencies were comparable in both BD patients and controls. The odds ratio for low copy number (<2 CN) was 0.6 (95% CI 0.30, 1.21, *P* = 0.16) and the odds ratio for high copy number (>2 CN) was 0.75 (96% CI 0.33, 1.73, *P* = 0.50). Therefore there was no evidence that either low or high *FCGR3B* was associated with BD, and in fact, both low CN and high CN were slightly decreased in BD patients relative to controls. Further, there was no evidence of associations between *FCGR3B* CN variants and clinical manifestations within BD patients as shown in Table 3.

## 4. Discussion

This is the first study to examine the relationship between *FCGR3B* CN variants and Behçet's disease, and we report no evidence of an association in terms of either disease susceptibility or clinical manifestations in this cohort of patients from Iran. However, a previous study of Turkish BD patients has reported associations between *FCGR2A*, *FCGR3A,* and *FCGR3B* SNPs and BD, in terms of both disease susceptibility and clinical manifestations [14], but this remains unconfirmed.

Other studies have reported intriguing but conflicting relationships between *FCGR3B* CN and vasculitis in the context of different diseases. For example, *FCGR3B* low CN (<2) is associated with Granulomatosis with Polyangiitis (Wegener's Granulomatosis) and Microscopic Polyangiitis (antineutrophil cytoplasmic antibody-associated systemic vasculitidies) in one study [8], and high *FCGR3B* CN (>2) in another [7], and no association has been observed with vasculitis in conjunction with systemic lupus erythematosus [15]. Further, similar to BD, susceptibility to other systemic vasculitidies including Giant Cell Arteritis and Kawasaki's disease has also been linked to SNPs within the FCGR gene cluster [16, 17].

The FCGR gene cluster is a complex genomic region, with both SNP and CNV polymorphism. While we were unable to demonstrate an association between *FCGR3B* CN and BD in this study, there are undoubted links to polymorphism in this region with vasculitic conditions. In future, the challenge will be to integrate CNV and SNP data into haplotypes in order to systematically evaluate and contrast associations with different vasculitidies. Such studies will provide valuable insight into the underlying pathogenetic mechanisms involved.

## Figures and Tables

**Table 1:** Baseline characteristics of patients with Behçet's Disease.

Characteristic	*N* (187)
Age at diagnosis (yrs), mean (±SD)	32.7 (8.2)
Female, *n* (%)	89 (48%)
Duration of symptoms at diagnosis (yrs), mean (±SD)	6.7 (6.1)
HLA testing	
HLA B51 positive	41/80 (51%)
HLA B5 positive	89/175 (51%)
Other tests	
Skin pathergy	101/107 (94%)
Symptoms	
Mucous membrane symptoms	187 (100%)
Skin symptoms	116 (62%)
Ocular symptoms	112 (60%)
Neurological symptoms	15 (8%)
Joint symptoms	58 (31%)
Gastrointestinal symptoms	13 (7%)
Vascular lesions	14 (7%)
ESR	
<20	83 (48%)
20–49	58 (34%)
≥50	32 (18%)

**Table 2:** Distribution of *FCGR3B* copy number (CN) variants in Behçet's (BD, *N* = 187) patients and controls (*N* = 178). When compared to 2 CN, there was no evidence that <2 CN or >2 CN genotypes were different in frequency between BD patients and controls (*P* = 0.16, 0.50, resp.).

*FCGR3B* CN	BD	Controls
1	15 (8.0%)	22 (12.4%)
2	161 (86.1%)	143 (80.3%)
3	10 (5.3%)	12 (6.7%)
4	1 (0.5%)	1 (0.6%)

Total	187	178

**Table 3:** Codistribution of FCGR3B copy number variation with clinical features within Behçet's patients.

		*FCGR3B* CNV			*N*	*P* ^1^
Clinical Parameter		<2	=2	>2		
Onset age: median (interquartile range)		26 (21,36)	26 (21,31)	25 (22,30)	187	0.67
Gender	Male: count (%)	8 (8.2%)	87 (88.8%)	3 (3.1%)	98	0.26
	Female: count (%)	7 (7.9%)	74 (83.1%)	8 (9.0%)	89	
HLA-B5	Neg: count (%)	8 (9.3%)	71 (82.6%)	7 (8.1%)	86	0.71
	Pos: count (%)	7 (7.9%)	78 (87.6%)	4 (4.5%)	89	
ESR	Normal: count (%)	9 (10.8)	70 (84.3%)	4 (4.8%)	83	0.40
	Elevated: count (%)	4 (4.4%)	80 (88.9%)	6 (6.7%)	90	
Genital apthosis^2^	No: count (%)	7 (10.8%)	53 (81.5%)	5 (7.7%)	65	0.79
	Yes: count (%)	8 (6.6%)	108 (88.5%)	6 (4.9%)	122	
Pseudofolliculitis	No: count (%)	10 (10.0%)	86 (86.0%)	4 (4.0%)	100	0.13
	Yes: count (%)	5 (5.8%)	75 (86.2%)	7 (8.1%)	87	
Erythema nodosum	No: count (%)	13 (9.4%)	116 (83.5%)	10 (7.2%)	139	0.98
	Yes: count (%)	2 (4.2%)	45 (93.8%)	1 (2.1%)	48	
Arthritis	No: count (%)	13 (8.9%)	126 (86.3%)	7 (4.8%)	146	0.17
	Yes: count (%)	2 (4.9%)	35 (85.4%)	4 (9.8%)	41	
Uveitis	No: count (%)	9 (8.3%)	91 (84.3%)	8 (7.4%)	108	0.61
	Yes: count (%)	6 (7.6%)	70 (88.6%)	3 (3.8%)	79	
Retinal vasculitis	No: count (%)	9 (7.5%)	104 (86.7%)	7 (5.8%)	120	0.81
	Yes: count (%)	6 (9.0%)	57 (85.1%)	4 (6.0%)	67	
Venous thrombosis	No: count (%)	15 (8.6%)	149 (85.6%)	10 (5.8%)	174	0.33
	Yes: count (%)	0 (0%)	12 (92.3%)	1 (7.7%)	13	

^1^Ordinal *P*  value (proportional odds ratio).

^2^All patients had oral apthosis.
